# Prolonged apnea during electroconvulsive therapy in monozygotic twins: case reports

**DOI:** 10.1186/1744-859X-10-30

**Published:** 2011-11-03

**Authors:** Maxim Zavorotnyy, Peter Zwanzger

**Affiliations:** 1 Mood and Anxiety Disorders Research Unit, Department of Psychiatry, University of Muenster, Muenster, Germany

## Abstract

In the present work, we report two cases of monozygotic twins who developed prolonged apnea during electroconvulsive therapy (ECT) as a complication of anesthesia. In both cases, prolonged action of succinylcholine caused by a butyrylcholinesterase (BCHE) deficiency was confirmed by means of the dibucaine number test. In both cases, genetic analysis using the polymerase chain reaction revealed haplotype combined A and K variant mutations of the BCHE gene, both in the heterozygous form. These data show the potential risk of BCHE deficiency as a complication of anesthesia during ECT, and in particular underline the possible genetic contribution within a complex pathogenetic model.

## Introduction

Electroconvulsive therapy (ECT) is considered to be the most powerful antidepressant intervention, and is thus recommended for the treatment of severe pharmacorefractory major depressive disorder (MDD) according to international guidelines. Moreover, ECT is usually well tolerated and the potential risks are mainly limited to anesthesia [1]. With regard to muscle relaxation during ECT, several reports of prolonged apnea due to the extended action of depolarizing neuromuscular blocking agents have been published of late [2-5].

Reduction of butyrylcholinesterase (BCHE) activity, as first described by German physician W Karow [6,7], can be induced by several somatic diseases, drugs such as acetylcholinesterase inhibitors, steroids, some antidepressants, oral contraceptives and organophosphate poisoning [4,8,9]. Also, inherited forms of BCHE deficiency are known [7], and affect up to 25% of the Caucasian population [5]. Mutations of the butyrylcholinesterase gene (BCHE; 3q26.1-3q26.2, OMIM 177400) were suspected in patients who experienced prolonged apnea after application of several neuromuscular blocking agents. The most common A (Asp70Gly) and K (Ala536Thr) variants were recently detected in patients with prolonged apnea during ECT [5].

Here, we report two cases of monozygotic twins, who both underwent ECT because of treatment-refractory severe major depressive episodes. Both patients showed prolonged apnea during anesthesia.

## Case presentation

### Patient 1

A 55-year-old, married male clerk was admitted for ECT in December 2006 because of medication-resistant psychotic depression according to *Diagnostic and Statistical Manual of Mental Disorders*, fourth edition (DSM-IV) criteria. After antidepressant medication was tapered off, ECT was delivered three times a week. Right unilateral (RUL) stimulation was administered using a Thymatron IV ECT apparatus (Somatics Inc., Lake Buff, IL, USA). During the first ECT, general anesthesia was carried out with thiopental; for muscle relaxation succinylcholine was given. During awakening prolonged apnea was observed for about 25 min and mask ventilation was required. Since BCHE deficiency was suspected, a dibucaine number test was carried out that showed an inhibition rate of 53% (normal range > 70%). A dose reduction of succinylcholine, switch to cisatracurium and prolonged mask ventilation allowed the continuation of ECT, which led to a full recovery (diagnosed on follow-up April 2011).

### Patient 2

A 58-year-old single weaver, the monozygotic twin of patient 1, was transferred to our hospital 2.5 years after our first patient's presentation because of severe MDD with psychotic features. In his case, his depressive symptomatology had already lasted 6 years without any response to medication. Similar to patient 1, ECT was chosen as treatment and anesthesia was carried out with etomidate and succinylcholine. After ECT, retarded awakening from anesthesia was observed, which lasted up to 3 h and persisted despite a switch to thiopental or propofol and a change of muscle relaxant to mivacurium. Prolonged apnea was observed only once and could be managed by mask ventilation so that no further changes were required and ECT could be administered until clinical remission was achieved. However, analysis of BCHE activity showed a marked deficiency as revealed by an inhibition rate of 43% on the dibucaine test. In this case, as with our first patient, the ECT continuation led to a stable remission, which had persisted on follow up in April 2011.

For the genetic analysis, PCR was carried out in both cases. A part of exon 2 and the whole of exon 4 including the adjacent 3' untranslated region of the BCHE gene were sequenced double-stranded from genomic DNA. The sequence alignment was conducted using the GenBank sequence NM_000055.2. Assessments with known mutations listed in the Human Gene Mutation Database (HGMD) revealed a haplotype combination of A and K variants of the BCHE gene, both in the heterozygous form.

## Conclusions

Taken together, these cases show the potential risk of BCHE deficiency as a complication of anesthesia during ECT and in particular highlight the genetic component as a relevant pathogenetic factor apart from somatic and pharmacological aspects. Early identification of inherited forms of BCHE deficiency seem thus to be especially important, since during a course of ECT, serial anesthesia is required for treatment. Finally, in view of the high heritability of major depression, patients with BCHE deficiency might also be informed since ECT might also be considered for treatment in other family members.

## Consent

Written informed consent was obtained from both patients for publication of this case report.

## Competing interests

The authors declare that they have no competing interests.

## Authors' contributions

Both authors were crucially involved in the treatment process described in the present work. MZ performed clinical follow-up investigations and drafted the manuscript. PZ critically revised the manuscript and gave his final approval for the version to be published.
